# Practical Prosthetic Castings1Read before First District Dental Society of New York, February 5, 1912.

**Published:** 1912-10-15

**Authors:** F. Ewing Roach

**Affiliations:** Chicago, Ills.


### PRACTICAL PROSTHETIC CASTINGS.[1]

BY F. EWING ROACH, D.D.S., CHICAGO, ILLS.

From a scientific viewpoint the subject of dental casting has been pretty thoroughly covered. The general technique is now quite familiar to all. The behavior and manipulation of waxes and investment compounds have been well discussed and, to most of us, this phase of the subject seems to be sufficiently well understood to warrant a feeling of satisfaction, so there seems to be but little need of directing your attention to this feature at present. But when we come to the construction of the great variety of prosthetic pieces we are confronted with a very different proposition.

A careful study of the situation has convinced the writer that the profession at large has not made the most of the casting process in prosthetic procedures. Dr. E. G. Coolidge of Chicago, has just completed a canvas of the members of the Illinois State Dental Society and he reports some interesting data regarding the status of the cast gold inlay in our state. He found that an average of 58 per cent. of the profession are using gold inlays, as against 42 per cent. who are using foil fillings. Similar data secured by your essayist show that a very much smaller per cent. have adapted the casting process to their prosthetic work. And while this splendid means of constructing our prosthetic pieces, such as crowns, bridges, splints, partial plates, etc., is far superior in many respects to all other methods, its advantages have, nevertheless, been overlooked by the great majority of the profession.

In my opinion all prosthetic pieces except the full denture can be made quicker and better by casting than in any other way. And when we say better we mean more accurate adaptation and anatomical reproduction, with greater fa-

[1]Read before First District Dental Society of New York, February 5, 1912.

cility and equal strength. The limitations of casting to this class of our work are the limitations of the dentist himself and not of the process.

Certain inherent physical difficulties surround the fitting and shaping of metals to the many intricate and irregular surfaces of the teeth and mouth by the swaging method that do not present themselves with the casting process. With castings it is a question of technique only.

If the profession has not made use of castings in prosthetic work to the same extent that it has for inlays, the question naturally arises, Why is this the case?

There are several factors that contribute to this condition. For a number of years prior to the introduction of the casting process we had studied the question of cavity preparation for inlays; and the permanency, as well as the many other questions regarding the inlay method of filling teeth, had been quite satisfactorily and favorably settled, so that we were in a very receptive mood, if you please, for the cast inlay. It came at a time when the whole dental world was ready to grasp it and make practical use of it at once. Had it been given to us ten or fifteen years earlier it would not have reached the same degree of popularity that it did in so short a time.

The variation in inlay technique, brought about by the casting process, was practically only a step, and that a very much simplified step, too, while in the prosthetic procedures we have had to make many, many changes in the details of our construction. We are constantly changing and improving our methods of doing this, that, or the other thing, until under the regime we will eventually arrive at the one best way of doing them all.

No two cases, of course, will call for exactly the same treatment, but similar methods will be followed for a certain class of cases. Our cases will be classified and our work systemized so that we will have a generally accepted method of doing each class. There is need for a better understanding of the difference in the physical characteristics of ROLLED

or *drawn* metals from those of the castings. We very frequently see bridges and partial plates of what I would term junk gold, using all the old crowns, bridges and the like, that accumulate in the office by melting them all together and without refining, cast them into some practical piece. This is a practice that cannot be too strongly condemned. Such castings are brittle and unless in large masses, will not withstand the strain that is likely to be brought to bear upon them.

An examination of the exhibits at the meeting of the Institute of Dental Pedagogics, in Chicago, last week, revealed the fact that the teaching of this subject is very meager. The prevailing objections to the teaching of casting methods in the dental schools were that student did not get the required training in the finger craft, and that the cost of materials was so much greater, that the management of the schools would not stand for the extra expense. Knowing that neither of these excuses occupied tenable ground, I made sufficient inquiry to satisfy myself that the teachers themselves had not worked out a system of technique in casting that they could offer as a substitute for the old methods. This should not be. The student should be taught modern, up-to-date dentistry. The methods in vogue five years ago are obsolete now, and it is an injustice to ourselves, and an imposition upon the public if we do not adapt this wonderful process to our prosthetic work.

It is a surprise to me to find so many who are still fitting bands, backing facings, and soldering them together; making the old two-piece gold crown with swaged cusps; bridges with dummies backed up and soldered individually to a swaged cusp to be subsequently assembled and soldered together.

In supplying a substitute for the natural tooth we should select methods and material that possess the greatest possibilities of reproducing the natural organ in appearance, strength and utility.

The successful crown should fulfill the following requirements: Good adaptation to root with reference to peripheral continuity and apposition to end of root; freedom from peridental impingement; close contact with approximating teeth, and at the same time preserving interproximate space gingivally; correct occlusion, anatomical alignment with adjacent teeth, adequate strength and compliance with the cosmetic requirements.

The cast base crown in my opinion, all things considered stands at the head of the list for the ten anterior teeth.

The technique of this crown is as follows: Trim root to gum, enlarge canal for suitable dowel, enlarging or extending orifice of canal labially and ligually, avoiding as much as possible weakening root by mesio-distal cutting. With the Roach Universal facer the root is trimmed labially below the gum by giving the facer a sweeping motion. A lingual interlocking step should be made by placing point of the flexible guard pin of the facer in the canal and forcing facer back until cutting edge is in line with the lingual periphery of root; then by holding it in one position, the lingual surface is cut down, leaving the inner portion next to the canal standing. A groove is cut from the orifice of the canal to the labial surface, the object of which is the reinforcement of wax at this point while fitting. It also adds stability to crown when set. Select any loose pin crown suitable for the case, grind to approximately fit root at labial margin but grind off its linguo-gingival angle. Fit dowel to canal, allowing it to project just enough so that it will reach bottom of hole in crown when labial end of same just touches end of root, thus determining exact length of dowel. Iridio-platinum wire 13 gauge threaded is preferred, but clasp gold may be employed. The dowel is heated and forced through a cone of wax and the porcelain crown is placed over end of dowel, wax softened over flame while being held between fingers to prevent overheating, and when soft forced partly to place on root, remove, trim off excess wax and repeat the above procedure until crown is fully seated and a perfect imprint of end of root is is obtained in the wax, then trim all excess of wax to peripheral outline of both tooth and root. The crown is removed and the dowel with its wax base pattern invested and cast preferable in 24-k. gold.

### PORCELAIN-FACED DOWEL CROWN.

While the porcelain-faced form of crown is seldom necessary, there are cases where it may be employed advantageously. The technique for this form of crown is as follows: Prepare root, fit band and dowel as for the usual construction, grind and fit facing in the mouth. The dowel is now removed and hammered flat on projecting end until wider than distance between facing pins. With knife-edge stone, file or plate punch make notches to correspond with pins. Facing and dowel in this relation are replaced in the mouth to verify the length of dowel, after which the dowel is threaded, heated and forced through a cone of inlay wax. Soften the cone of wax and adjust facing and dowel with wax cone and force them to place on the root, having band in place at the time. Chill the wax and remove all parts together by passing an instrument under the band and working off carefully from side to side so as not to disarrange the parts. Trim off excess, remove facing, put carbon points in pin holes, and you are ready to cast and finish in your favorite way.

For this class of work there is no objection to the use of scrap and junk gold for the casting—preference is given iridio-platinum for dowel and 22 or 24-k. gold for band. The facing pins may be bent down at an angle for 45′ or 50′ in cases of close bite.

### GOLD CROWN.

Some dentists have adopted the cast cusp gold crown, but the technique of this crown is so little understood that its advantages over other methods of construction are not appreciated. To my way of thinking, there is no other procedure that compares with it in any respect. The facility with which contour and occlusion may be produced is a marvel of simplicity and accuracy.

We frequently hear the claim made that this crown requires too much gold to permit of its general use. This objection I am prepared to prove is not correct. The technique of this crown is simple, quick and accurate, and needs but a few words to explain.

With tooth properly prepared, the band is fitted in the usual manner and contoured with pliers. Band should be made long enough so that it may be cut and fitted to approximate the occlusion. A cone of inlay wax is now softened and placed over occlusal end of band and patient instructed to close upon it. Chill the wax, remove together with the band, carve to anatomical form indicated, and with Suction Wax Carver remove any excess of wax from inside of crown. For this purpose I prefer the more transparent waxes so that the thickness may be more readily determined by holding up to the light while carving. Scrap gold may be used for this class of work to better advantage than for any other purpose.

### SPLIT ROOT.

While the casting process has completely revolutionized all my prosthetic and operative procedures in everyday routine work, it has at the same time enlarged the field of reparative measures so that operations heretofore practically impossible of satisfactory accomplishment have become commonplace and simple, with results beyond my fondest hopes. As an illustration of this I am going to give you a description of a method of repairing split roots that has given me great satisfaction.

If our good friend Taggart had done nothing more than made possible the restoration to comfort and usefulness of teeth with split roots he would have rendered a great service to humanity. And while this operation is only one of many, it is unique in character and shows the possibilities of the process.

When a case presents itself with root fractured not more than one-half its length and the fractured piece is intact the repair is simple and positive. The procedure is as follows: Draw fractured part into close apposition by twisting wire around it. The steps outlined above for cast base crown are identical, so that when wax base is secured the piece of root is extracted and carefully placed in position on wax base—the groove across end of fractured piece and the side of canal affords a definite guide in locating it. The piece is now secured to the crown and wax base with wax. The fractured surface and dowel is coated with a vegetable oil and a batch of cement, mixed stiff, is pressed into apposition with fractured surface of root. When cement is hard it is scraped down flush with piece of root, after which it is separated from crown and the piece of root, which is carefully separated from wax base and crown, the wax base is then readjusted to its proper position on the cement model, and inlay wax melted into space originally occupied by piece of root, and scraped down flush with surface of cement model. This gives us an exact reproduction in wax of the piece of root, united as an integral part of the original wax base in its proper relative position, which is also true of the casting. When cementing such a crown to place in the mouth I prefer to use Evans' gutta percha cement on fractured surface.

In my experience with casting I have made the following observations:

That nearly all prosthetic castings should be made in combination with iridio-platinum or gold in wire or plate form as a means of reinforcement.

That iridio-platinum on account of its great strength and freedom from oxidation affords the best reinforcement.

That 24k. gold reinforced with iridio-platinum is the best for inlay abutments.

That the reinforcement plan expedites as well as strengthens the work and obviates bulkiness, which is so essential in many instances.

That it is best not to heat any alloy of gold containing base metals to the point of oxidation when casting upon it.

That it is unnecessary and detrimental to heat a flask to a red heat or anywhere near it when burning out wax.

That the elastic limit of scrap gold is practically nil and it should not be used where much strain will be brought to bear upon it.

That alloys of gold with platinum will become very brittle when cast a few times. This Dr. Taggart tells us is due to contamination with silica contained in the investment.

That the casting process makes possible the employment of almost all forms of porcelain teeth and that provision should be made for cementation rather than casting directly on to the procelain.

That nearly all inlay abutments, regardless of size and shape of cavity, should have some form of supplemental pin anchorage.—*Journal of Allied Societies.*

